# Food Safety Knowledge and Practices of Pregnant Women and Postpartum Mothers in Slovenia

**DOI:** 10.3390/foods10102412

**Published:** 2021-10-12

**Authors:** Mojca Jevšnik, Anja Česen, Marina Šantić, Andrej Ovca

**Affiliations:** 1 Faculty of Health Sciences, University of Ljubljana, Zdravstvena pot 5, 1000 Ljubljana, Slovenia; mojca.jevsnik@zf.uni-lj.si (M.J.); anjacesen.5@gmail.com (A.Č.); 2 Faculty of Medicine, University of Rijeka, Braće Branchetta 20/1, 51000 Rijeka, Croatia; marina.santic@medri.uniri.hr

**Keywords:** pregnancy, postpartum, food safety, food handling, expressed breast milk, infant formula

## Abstract

Food safety during pregnancy and postpartum is important for preventing foodborne diseases, while pregnant women are considered vulnerable due to their immunomodulatory condition. The current study aimed to investigate the self-reported food safety knowledge and practices of pregnant women and postpartum mothers in Slovenia using an online questionnaire and to compare the results with nonpregnant women as a control group. The study was conducted with 426 women, of whom 145 were pregnant, 191 were not pregnant, and 90 were postpartum. The online questionnaire consisted of questions related to food safety risk perception, hand hygiene, food purchase, food storage, food preparation and handling of infant formula and breast milk. The results showed that women generally have basic knowledge of proper food handling and are aware of food safety, but some specific gaps were identified in food handling at home, especially concerning microbiological risks. However, the results showed that pregnant women performed better than the postpartum group, and both groups performed significantly better than the nonpregnant group. The media was most frequently cited as a source of food safety information, especially by the pregnant group. Trained health workers should also inform women on how to ensure food safety in the home environment.

## 1. Introduction

Epidemiological data [1] and consumer studies have shown that a significant proportion of foodborne diseases (FBD) arise from inappropriate practices in the home environment. Pregnant women are considered a vulnerable group for FBD due to their immunomodulatory condition. Therefore, it is important that they particularly avoid foods with a high risk of infection [2,3]. Ensuring food safety is important for preventing FBDs such as listeriosis and toxoplasmosis [4]. Listeriosis and toxoplasmosis have harmful effects on pregnant women and even more on foetuses as they can cause miscarriage or stillbirth. Detection and treatment of both infections can significantly change the outcome for infected women, foetuses and new-borns [5].

Listeriosis is the third leading cause of death from foodborne pathogens in the U.S.A [6]. Between 2005 and 2014, 80 cases of listeriosis were reported in Slovenia, of which 18 resulted in death [7,8]. In 2017, listeriosis was confirmed in 13 people, and one person died [8]. Pregnant women are among the group at risk for active *Listeria* infection due to the weakening of their cellular immune system during pregnancy and the ability of the bacterium to bind to cellular receptors in the placenta [9]. The CDC [10] estimates that listeriosis occurs in 14% of pregnant women each year. The incidence of listeriosis during pregnancy varies from one ethnic group to another, depending on their habits and food handling, socioeconomic status, and place of residence [11,12,13]. Compared to healthy people, susceptibility to listeriosis is 13–20 times higher in pregnant women [14].

In a study by Silk et al. [15], 29% of *Listeria* infections in pregnant women resulted in miscarriage or neonatal death. Broadly similar results were obtained by Awofisayo et al. [16], who reported that 22% of pregnancies affected by *Listeria* infection ended in stillbirth or miscarriage. To reduce the possibility of *Listeria* infection, pregnant women are advised to avoid eating foods considered high risk for *Listeria* and to follow general advice on proper food handling [17]. Foods most commonly associated with *Listeria* contamination include unpasteurised (raw) dairy products, soft cheeses made from unpasteurised milk (e.g., Feta, Brie, Camembert), cheeses with noble moulds, raw meats, meats that have been improperly heat-treated, hot dogs, meat spreads, pies, unwashed raw vegetables and fruits, raw and smoked seafood [10,14,17,18]. Maia et al. [19] emphasises education and information about the consumption and handling of risky foods associated with listeriosis as a key measure to reduce exposure to *Listeria* and state that it should be targeted primarily to at-risk groups. Despite numerous educational campaigns and media attention due to several fatal outbreaks, many authors report that awareness of listeriosis among at-risk individuals is low [20,21,22]. Xu et al. [23] found that of 78.9% of pregnant women who reported being aware of *Listeria*, only 28.9% reported not consuming high-risk foods associated with *Listeria*.

*Toxoplasma gondii* is a parasite that infects one third of the world’s population [24]. Humans are typically infected through three main routes of transmission: foodborne infection, animal-to-human transmission (zoonoses), and transmission from a pregnant woman to the foetus [25]. In 2017, 24 cases of toxoplasmosis were reported in Slovenia, which is 11 fewer than in 2015 [8]. The risk of infection during pregnancy is higher due to the modulated immune system and can lead to severe pathological changes or neurological damage to various organs [5]. A woman can become infected with *Toxoplasma gondii* by eating unpasteurised milk, raw vegetables, undercooked meat (especially pork, lamb, and venison), shellfish, and food contaminated with knives and cutting boards that have been in contact with raw meat or shellfish. Infection can also be caused by drinking contaminated water, after accidental ingestion of the parasite when in contact with cat faeces and, in rare cases, through transplantation of infected organs or blood transfusion [5,24,25,26].

A woman who has not been previously exposed to *Toxoplasma* infection and becomes infected for the first time during pregnancy may transmit the infection vertically to the foetus [26,27]. Thus, the *Toxoplasma* parasite is transmitted through the placenta into the bloodstream of the foetus. Toxoplasmosis in pregnancy can lead to miscarriage, premature birth or stillbirth [28].

In Slovenia, testing pregnant women for toxoplasmosis has been required by law since 1995. The first test should be performed as early as possible: at the first check-up between the 10th and 12th week of pregnancy. Pregnant women with a negative result are tested again at the 20th week and around the 30th to 34th week of pregnancy [29]. Jones et al. [30] found that knowledge about infections, except for the risk of transmission of *Toxoplasma* by cats, is low. Only 30% of women are aware that *Toxoplasma* infection can be acquired through food. Therefore, to prevent toxoplasmosis, the importance of proper food handling is emphasised [24,25].

Pregnancy is a time when women are more motivated to learn about ensuring food safety because they are concerned about their health and that of the child. Many organisations emphasise the importance of prevention and educating pregnant women about food safety and eating low-risk foods [2,9,14,31,32,33]. In Slovenia, organised education of pregnant women about healthy eating takes place in parenting classes, in which midwives are also actively involved. Although studies focusing on knowledge and awareness of specific pathogens relevant during pregnancy (*Toxoplasma gondii* and *Listeria monocytogenes*) can be found in the scientific literature [12,23,30,34], studies on perceptions of other food safety risks, food handling knowledge and food handling practices during pregnancy [35,36] and in the postpartum period [37] are extremely underrepresented. Although individual studies have previously compared pregnant and non-pregnant women [36] or pregnant women and mothers [37], we have not found a study comparing non pregnant women, pregnant women and postpartum mothers in this context. In addition to the need to fill the gap in the literature, the question arises as to whether it makes sense to include a food safety programme into such parenting preparation courses, as their number of participants decreases year by year [38]. The current study aimed to (i) investigate the self-reported food safety knowledge and practices of pregnant women and postpartum mothers using an online questionnaire and (ii) compare the results with nonpregnant women as a control group, and (iii) compare the results with the results of a similar study by Guneri et al. [35] and an earlier study in Slovenia by Jevšnik et al. [36].

## 2. Materials and Methods

### 2.1. Research Protocol

The cross-sectional data for this study were collected with an anonymous online questionnaire administered for a three-month period from 27 May until 27 August 2019. The target population was pregnant women and postpartum (up to 6 months) mothers. Initially, a linear snowball sampling approach with a convenience sample of the initial subject was applied [39]. Because of the relatively low response among the target population, the questionnaire was also delivered to closed groups with pregnant women and mothers after childbirth as members on the social networks. The Chair of Midwifery at the Faculty of Health Sciences approved the research protocol.

All respondents were anonymised and participated voluntarily. During the introduction, the respondents were informed that the data would be collected and analysed anonymously exclusively for research study purposes. In total, 898 women responded to the invitation (clicked on the questionaries’ link), whereby 426 (145 pregnant women, 191 nonpregnant women and 90 postpartum mothers) completed the questionnaire and were included in data amylases.

### 2.2. Research Instrument—Questionnaire

The online questionnaire was prepared based on the questionnaire developed by Guneri et al. [35]. The official permission to use and adapt the original questionnaire was granted by their authors on 24 December 2018. The original questionnaire [35] was adapted to examine general aspects of food safety, regardless of geographic location. Some additional questions were added to consider a previous Slovenian study [36] and postpartum mothers [37]. The evaluation of the content of the questionnaire was done by the first and last author of this study, who are experienced in food hygiene and knowledgeable about food safety among consumers. Considering the target population, a midwifery expert was also consulted. The questions can be divided into seven thematic sections: Demographic characteristics, food safety risk perception, hand hygiene, food purchase, food storage, food preparation and handling of infant formula and breast milk. The last set was offered only to postpartum subgroup.

The estimated duration for completing the questionnaire was 5 min and 47 s. The questionnaire was pilot tested by 11 women (6 pregnant and 5 nonpregnant) to determine the clarity of the question and gauge the length of time necessary to answer the survey. The questionnaire was revised accordingly. The final online questionnaire consisted of 40 questions, of which 35 were closed-ended, two were semi-closed, and three were open-ended. The final version of the questionnaire is available as Supplementary Materials.

### 2.3. Data Analysis

The data were evaluated and analysed using the Statistical Program for the Social Sciences (SPSS, Version 25.0, Chicago, IL, USA, 2006). To examine the relationships among and between the variables, a chi-square test for independence (χ^2^ test) for categorical variables (nominal and dichotomous types) and an independent sample t-test for ordinal variables (5-point Likert-type measurement scale) treated as a continuous variable were used.

## 3. Results and Discussion

### 3.1. Demographic Characteristics

The demographic data (Table 1) show that overall the respondents are balanced in terms of age, education and place of residence. Although the nonpregnant respondents are younger and less educated compared to the other respondents (Table 1), there is no significant difference between the subgroups of pregnant and postpartum respondents in this regard; 18.1% of all respondents reported having experienced food poisoning. The rest reported they had not (65.7%) or were unsure about it (16.2%), while there is no significant difference in their maternity status.

### 3.2. Food Safety Risk

Because pregnant women are at increased risk of foodborne illness, it is important to pay attention to food safety during pregnancy and avoid foods that are at increased risk of infection with *L. monocytogenes* and *T. gondii* (e.g., unpasteurised dairy products, soft cheeses made from unpasteurised milk, raw meat, etc.) [14]. The results in Table 2 show that the pregnant and postpartum women have a higher awareness of food safety risks than the nonpregnant respondents do, while there is no significant difference between the pregnant and postpartum subgroups.

The rate of consent to eat risky food during pregnancy is similar. Consequently, nonpregnant respondents are more likely to report preparing these types of dishes than the pregnant and postpartum group, and the postpartum group is more likely than the pregnant group, although all of them prepare this kind of dishes occasionally. Similar results were obtained earlier by Jevšnik et al. [36], who found that pregnant women were less likely to prepare and enjoy high-risk meals (e.g., pastries and cakes with raw eggs, raw meat dishes, and raw fish) compared to nonpregnant women.

While there are no significant differences in the knowledge of specific microorganisms (*Salmonella*, *Listeria*, *Campylobacter*), the parasite *T. gondii* is significantly better known (*p* < 0.001) in the pregnant and postpartum group (61.9%) compared to the nonpregnant group (38.1%). Jones et al. [30] also found that 58% of pregnant women are aware of the increased likelihood of infection with the parasite *T. gondii*, while Mateus et al. [12] state that 81.3% of pregnant women have heard of toxoplasmosis.

The higher awareness of pregnant and postpartum respondents of infection with *T. gondii* in the current study can be attributed mainly to the legally required screening of all pregnant women in Slovenia for toxoplasmosis [29]. Lower awareness is observed in relation to *Listeria*, where only slightly more than half (Table 2) of pregnant respondents are aware of it, and one third (34.5%) admit that they do not know if there is an increased risk of infection during pregnancy from *Listeria*. In a study among Italian pregnant women and young women after birth, Ricchi et al. [40] estimated that 94% of pregnant women know *T. gondii*, while 39.5% are unfamiliar with *L. monocytogenes*.

Media (TV, radio, magazines) were mentioned most frequently (63.8%) as a source of food safety information, followed by the Slovenian Consumer Association (36.9%), doctors (26.3%), recommendations from retailers when shopping (26.1%), parenting class (23.9%), midwives (11.0%). However, media is more frequently (*p* = 0.011) reported by pregnant respondents (67.8%) than the postpartum subgroup, indicating intensive information seeking during pregnancy. In contrast, midwives are more frequently (*p* = 0.038) reported by the postpartum subgroup (54.5%) compared to the pregnant subgroup (45.5%). Jevšnik et al. [36] reported that the majority of pregnant respondents received information about food safety through mass media, and only 14.2% in a parenting class or from health professionals. Similar results were obtained by Guneri et al. [35]. They found that more than half of pregnant women (62.3%) did not receive food safety education and more than half of those who received education received it through television.

It is evident that pregnant women, compared to a previous study [36], still receive most food safety information through various mass media, the relevance of which may be questionable. This may result from healthcare professionals not paying enough attention to food safety, so pregnant women seek information through mass media [41]. Taylor et al. [34] reported that health workers have an important informative role during women’s pregnancy as participants viewed health workers as a reliable and essential source. Among the latter, midwives are especially important because of their direct contact with pregnant and postpartum women.

### 3.3. Hand Hygiene

While there is no influence of maternity status on the recognition of occasions requiring hand washing and the washing technique itself, the pregnant group most frequently recognises gastrointestinal diseases as a consequence of not washing hands after using the toilet (Table 3). In the previous study [36], pregnant women reported washing their hands more frequently after performing dirty tasks (e.g., after handling eggs) than the nonpregnant group did.

As noted by Byrd-Brebenner et al. [42], hands are an important vehicle for the spread of pathogens in the kitchen, and proper handwashing is critical to avoid cross-contamination. In our study and the study by Guneri et al. [35], most pregnant women correctly answered the question in which cases they should wash their hands. However, the difference in knowledge was evident in the question “How do we wash our hands properly and effectively before we start preparing food?” to which our respondents answered mostly (88.3%) correctly, while the majority (68.3%) of pregnant women in Guneri et al. [35] study answered this question incorrectly.

Trepka et al. [37] found that 67.6% of parents always wash their hands with soap and running water after changing their baby’s diaper. However, in our study, the percentage was lower as only 41.1% of postpartum mothers always (31.1% almost always) wash their hands with soap and running water after diaper changes. The rest do it less often.

It is a matter of concern that before preparing baby food or breastfeeding, 36.7% reported always washing their hands with warm water and soap, which is different from the figures reported by Stanonik [43] and Trepka et al. [37], where 64% of parents and 75.2% of mothers wash their hands with soap and running water before preparing baby food for their infant.

### 3.4. Food Purchase

In the current study, the condition of the packaging receives the greatest attention, and the refrigeration temperature receives the least attention from the respondents when buying food (Table 4).

Except for declaration and refrigeration temperature, pregnant respondents pay the greatest attention compared (not significant) to the other two subgroups (Table 4). When asked how often they pay attention to the temperature of the refrigerator/freezer in the store while shopping, more than half of the respondents (60.7% of pregnant, 58.1% of nonpregnant, and 54.4% of postpartum respondents) answered “never”. On the other hand, almost half of the respondents (47.6% of pregnant, 41.9% of nonpregnant and 42.2% of postpartum respondents) answered that they always look for undamaged packaging when buying food. In a previous study, Jevšnik et al. [36] found that pregnant women were more cautious than the nonpregnant group when purchasing food, as they were more likely to check the best-before date and packaging for possible damage.

When asked further about the best place to buy safe milk, the group of pregnant and postpartum respondents were much more in favour (*p* < 0.001) of buying sterilised or pasteurised milk at grocery stores (61.7%) than nonpregnant women (42.9%). The latter are not hesitant to buy fresh milk even from milk vending machines or farms or think it makes no difference where they buy milk. The pregnant subgroup is more cautious in this regard (67.6%) than the postpartum group (52.2%) (*p* = 0.019). Guneri et al. [35] established that 91.7% of pregnant women gave an incorrect answer to the question of where to buy safe milk. A previous Slovenian study [36] found that women who lived in villages and were under 35 years of age were more likely to buy various foods directly from farmers without knowing whether the food had been tested, as domestic food production is a respected family tradition in Slovenia.

They were also asked what is not important from the food safety point of view when buying canned food. Respondents could choose among the following answers: brand, vacuum packaging, packaging condition and durability date. In total, 81% choose the brand with no significant differences between subgroups.

### 3.5. Food Storage

Most respondents fully agree that food and cleaning products cannot be stored in the same place (97.7%) and that milk and dairy products spoil fastest at room temperature (95.5%) compared to fruits and vegetables, cereals and legumes. The compliance rate (%) regarding proper food storage is generally higher in the pregnant and postpartum groups than in the nonpregnant group (Table 5).

The largest and significant difference is observed in the context of avoiding cross-contamination in the storage of cooked and raw foods, where the nonpregnant indicate more risk practices (Table 5). When considering the storage location of milk, the postpartum group differs significantly from the other two subgroups (Table 5). Sampers et al. [44] established that 20% of their respondents do not know what the temperature is in a home refrigerator. Jevšnik et al. [45] reported that half of Slovenian consumers do not know the temperature in their household refrigerators. Jevšnik et al. [36] previously found that nonpregnant women check the temperature in their home refrigerator more frequently (*p* < 0.001) than pregnant women did, as 32.7% of pregnant women never or rarely checked the temperature in their home refrigerator.

In the current study, the majority (79.1% of nonpregnant, 79.3% of pregnant women, and 81.1% of postpartum respondents) stated that they had never measured the refrigerator temperature. When further asked what they thought the best temperature in the refrigerator was, the responses resulted in a mean of 6.19 °C (SD = 3.07). The non-pregnant group reported slightly higher values (6.22 °C; SD = 3.36) when compared to the pregnant (6.16 °C; SD = 3.08) or postpartum subgroups (6.17 °C; SD = 2.33).

Those who reported measuring the temperature in their household refrigerator reported an average temperature of 5.83 °C (SD = 2.45), with postpartum respondents reporting the lowest average temperature of 5.53 °C (SD = 1.74) when compared to the pregnant (5.73 °C; SD = 2.80) or nonpregnant subgroup (6.03 °C; SD = 2.45). The reported temperatures are consistent with a recent study among Slovenian consumers with a 24-h measurement in 50 households, where the average internal temperature of the standardised test product was 5.95 °C (SD = 2.24), and the refrigerator air temperature was 5.91 °C (SD = 2.23) [46]. However, it is worth noting that 50% of the studied refrigerators allowed an average internal temperature above 6 °C during the 24-h temperature measurement and that temperature displays among the studied refrigerators were present only in 16%, while control thermometers were not observed at all [46].

### 3.6. Food Preparation

In a review, Byrd-Brebenner et al. [42] reported that 14% of all FBD in the UK might be due to inadequately cleaned cutting boards and knives. They also summarised the results of other consumer surveillance studies and found that the vast majority of consumers do not clean cutting boards and utensils adequately to avoid cross-contamination. Self-reported practices in the current study show selective prevention of cross-contamination by cutting boards. While the frequency is highest when washing the cutting board after cutting raw meat and before cutting heat-treated meat, we can observe the lowest frequency when separating the cutting board for raw meat and raw vegetables (Table 6). Compared to the results of the previous study [36], differences in mean scores in our study showed that pregnant women find it more important than nonpregnant women did to separate cutting boards for raw foods from boards for cooked foods.

We can also observe that the frequency in the groups of pregnant and postpartum women is generally higher than in the group of nonpregnant women, with the highest number among the postpartum subgroup (Table 6). The largest and significant difference is observed in relation to hand washing after handling eggs, where the nonpregnant women report a riskier practice (Table 6). Similar results were obtained in the study by Jevšnik et al. [36], with differences in mean scores showing that pregnant women believe more strongly than the nonpregnant group in washing their hands thoroughly after handling eggs.

When further asked if they agreed with keeping raw meat and raw fish separate in the refrigerator, the pregnant and postpartum subgroups agreed with this statement less (11.1%) than the nonpregnant subgroup (16.2%) (*p* = 0.035); 51.1% of the pregnant and postpartum subgroup and 39.8% of the nonpregnant subgroup disagreed. The remainder were not sure. In the study by Guneri et al. [35], 73.6% of pregnant women indicated that it is important to keep meat and fish separate.

Thawing raw foods on the kitchen counter was the most commonly reported practice (76.5%) among all respondents, but it was reported more frequently by the nonpregnant subgroup (81.2%) compared to the pregnant and postpartum subgroups (72.8%) (*p* = 0.042). However, the majority of all respondents (90.8%) fully agree that thawed meat should not be refrozen. The results are similar to Sterniša et al. [47] in Guneri et al. [35], who reported that the majority (73%) of respondents and 76.3% of pregnant women, respectively) apply inappropriate forms of thawing frozen food at room temperature. In addition, Jevšnik et al. [36] found that pregnant women are significantly more likely than nonpregnant women to thaw frozen food on the kitchen counter. Proper thawing of food (in the refrigerator, under cold running water, or in the microwave) is an important element in ensuring food safety, as improper thawing affects the growth and development of harmful microorganisms [42,48].

### 3.7. Preparation and Handling of Infant Formula and Breast Milk

Although infections from infant formula or breast milk are rare, powdered food, as well as breast pumps, can be contaminated with harmful bacteria. This can be particularly serious in the first two months of life and in premature, immunocompromised, or low-birth-weight infants [49].

Respondents in the postpartum subgroup were, therefore, additionally asked about their practices that might affect the health of their new-borns. The majority (48.9%) always let the water boil for two minutes before cooling it and prepare formula for the baby, while 27.8% never do. Trepka et al. [37] found that 62.1% of parents always boil water for at least two minutes before cooling and mixing it with infant formula, while 15.8% of parents never boil water for this long. Also, 83.3% never leave prepared formula or bottled breast milk at room temperature for more than two hours. The rest sometimes (11.1%), occasionally (2.2%) or always (3.3%) do this. The latter is comparable to a study conducted in Florida, where 79.4% of parents never leave formula or expressed breast milk outside the refrigerator for more than two hours [42].

The majority (83.3%) agreed that unconsumed breast milk should be disposed of, while 4.4% did not or did not know (12.2%). Stanonik [43] came to a similar conclusion, finding that 75% of parents immediately throw away the rest of the formula that the child did not consume. Pumped breast milk in our study is stored in plastic containers (52.2%), glass containers (33.3%) or plastic bags (14.4%).

## 4. Conclusions

Pregnant women and postpartum mothers are clearly aware of the high risk of infection with *T. gondii* during pregnancy compared with nonpregnant women, whereas the difference in risk perception is less clear with respect to *L. monocytogenes*. Results showed that women generally have basic knowledge on proper food handling and are aware of food safety, but some specific gaps were found in food handling at home, especially regarding microbiological risks (e.g., avoiding cross-contamination in the storage of cooked and raw foods, thawing raw foods on the kitchen counter, washing hands after handling raw eggs and before preparing baby food or breastfeeding).

In general pregnant women performed better than the postpartum group, and both groups performed significantly better than the non-pregnant group. The results also show that pregnant women are attentive to the analysed food safety topics. However, only a small proportion of pregnant women and the postpartum group received food safety information in parenting classes or by health professionals whereas the media was most frequently cited as a source of food safety information, especially by the pregnant group. The latter suggests that no progress has been made in this area since the previous national study.

Too little attention is paid to food safety topics being tailored to the specific target group. There is a need to develop tailored educational materials to be made available to pregnant women in prenatal parenting classes or gynecology clinics. However, evaluation of the impact of these materials should not be neglected. Informing pregnant women and postpartum mothers about food safety issues through trained health professionals (e.g., midwives) with credible resources is crucial for the consistency of messages and increasing food safety awareness among vulnerable consumer groups.

## 5. Research Limitation

A questionnaire is an appropriate tool for conducting studies on larger samples, but it has weaknesses, especially in self-reported practices that may be susceptible to respondent bias. In addition, the results obtained from the questionnaire cannot be generalised to the whole population because of the relatively small sample. A larger realisation of the sample would likely be achieved by surveying women in gynaecology clinics throughout the country, where all three groups of women could be included: pregnant women, nonpregnant women, and postpartum mothers. Validation of the questionnaire would further increase the reliability of the results.

## Figures and Tables

**Table 1:** Demographic data of surveyed population (*n* = 426).

	Maternity Status						Total	
	Nonpregnant		Pregnant		Postpartum (≤6 Months)			
	*n*	%	*n*	%	*n*	%	*n*	%
**Age**								
≤25	142	74.3	40	27.6	27	30.0	209	49.1
26≤	49	25.7	105	72.4	63	70.0	217	50.9
**Education**								
Finished primary or secondary school	122	63.9	55	37.9	39	43.4	216	50.7
Higher education	69	36.1	90	62.1	51	56.7	210	49.3
**Place of residence**								
Town or suburb area	80	51.0	74	51.0	35	38.9	189	44.4
Village	111	49.0	71	49.0	55	61.1	237	55.6

**Table 2:** Agreement rate (%) when considering the risk of infection by specific microorganisms or foods during pregnancy by maternity status.

	Maternity Status			*p*	
	Nonpregnant	Pregnant	Postpartum (≤6 Months)	NP vs. P & PP	P vs. PP
Is there an increased risk for infection during pregnancy because of…					
*Listeria monocytogenes*	47.6	55.9	60.0	**0.022**	0.823
*Toxoplasma gondii*	60.7	77.9	78.9	**0.000**	0.970
Is it safe to eat during pregnancy…					
RTE meals including raw meat	3.1	0.0	1.1	**0.000**	0.086
Pastries or cakes containing raw eggs	5.2	0.7	1.1	**0.000**	0.560
Raw fish	2.6	0.0	2.2	**0.000**	0.115
Chateau	4.7	2.8	2.2	**0.001**	0.585
Eggs with liquid yolk	26.2	21.4	24.4	**0.005**	0.468

RTE—Ready to eat; *p*—association between maternity status and answers to the question; vs.—versus; NP—not pregnant; P—Pregnant; PP—Postpartum.

**Table 3:** Compliance rate (%) in hand hygiene occasions, hand hygiene technique and hand-borne diseases after toilet use by maternity status.

	Maternity Status			*p*	
	Nonpregnant	Pregnant	Postpartum (≤6 Months)	NP vs. P & PP	P vs. PP
Hand hygiene occasions	97.4	97.9	97.8	0.740	0.937
Hand hygiene technique	90.1	88.3	93.3	0.956	0.205
Hand-borne diseases after toilet use	82.7	93.1	84.4	**0.033**	0.033

*p*—association between maternity status and answers to the question; vs.—versus; NP—not pregnant; P—Pregnant; PP—Postpartum.

**Table 4:** Frequency (arithmetic mean) of checking food characteristics, storage and hygiene conditions when purchasing food by maternity status, listed from highest to lowest overall arithmetic mean.

	Maternity Status						*p*	
	Nonpregnant		Pregnant		Postpartum (≤6 Months)		NP vs. P & PP	P vs. PP
	X	SD	X	SD	X	SD		
Condition of packaging	3.95	1.11	4.08	1.04	3.93	1.17	0.498	0.309
Durability date	3.77	1.12	3.89	1.14	3.69	1.16	0.697	0.194
Orderliness of the seller	3.26	1.16	3.18	1.19	2.93	1.23	0.127	0.129
Hygiene of service	2.89	1.15	2.96	1.37	2.63	1.29	0.677	0.072
Storage conditions	2.86	1.23	2.88	1.32	2.62	1.34	0.523	0.155
Origin	2.79	1.14	2.87	1.11	2.66	1.08	0.986	0.149
Declaration	2.84	1.14	2.80	1.08	2.42	1.09	0.086	**0.010**
Content of vitamins and minerals	2.14	1.02	2.42	1.02	2.06	1.09	0.775	0.187
Refrigeration temperature	1.76	1.10	1.70	1.07	1.87	1.16	0.980	0.253

*p*—association between maternity status and answers to the question; vs.—versus; X—arithmetic mean; SD—Standard deviation; 1—Never; 2—Sometimes, 3—Usually; 4—Almost always; 5—Always; NP—not pregnant; P—Pregnant; PP—Postpartum.

**Table 5:** Compliance rate (%) regarding food storage practices by maternity status.

	Maternity Status			*p*	
	Nonpregnant	Pregnant	Postpartum (≤6 Months)	NP vs. P & PP	P vs. PP
Milk should be stored on the refrigerator door.	27.2	23.4	42.2	0.441	**0.002**
Cooked food should be stored at room temperature until cooled down.	67.5	73.8	76.6	0.094	0.621
Hard-boiled eggs can be stored at room temperature for more than two days.	23.6	17.2	22.2	0.267	0.346
Cooked and raw foods should be stored separately.	53.4	69.7	73.3	**0.000**	0.546
Freezing does not kill bacteria but only stops them from multiplying.	59.7	62.8	68.9	0.250	0.338
For storage or heat treatment, important instructions are written on the packaging.	72.3	74.5	81.1	0.259	0.240

*p*—association between maternity status and answers to the question; vs.—versus; NP—not pregnant; P—Pregnant; PP—Postpartum.

**Table 6:** Frequency (arithmetic mean) of self-reported practices related to cross-contamination prevention by maternity status, listed from highest to lowest overall mean.

	Maternity Status						*p*	
	Nonpregnant		Pregnant		Postpartum (≤6 Months)		NP vs. P & PP	P vs. PP
	X	SD	X	SD	X	SD		
Washing the cutting board after cutting raw and before cutting heat-treated meat.	4.51	0.83	4.64	0.78	4.72	0.70	**0.039**	0.423
Washing dishes with detergent or in the dishwasher before re-use.	4.28	1.03	4.37	0.96	4.61	0.73	0.052	**0.033**
Washing hands after handling eggs.	3.95	1.11	4.13	1.06	4.27	1.09	**0.027**	0.345
Separating cutting boards for raw and cooked food.	3.82	1.15	3.88	1.13	4.08	1.17	0.240	0.206
Washing salad in the kitchen sink before handling raw meat.	3.78	1.28	3.80	1.17	3.68	1.40	0.828	0.489
Having separate cutting boards for raw meat and raw vegetables.	3.72	1.37	3.77	1.34	3.79	1.41	0.674	0.929

*p*—association between maternity status and answers to the question; vs.—versus; X—arithmetic mean; SD—Standard deviation; 1—Never; 2—Sometimes, 3—Usually; 4—Almost always; 5—Always; NP—not pregnant; P—Pregnant; PP—Postpartum.

## Data Availability

The data presented in this study are available on request from the corresponding author.

## Supplementary Materials

The questionnaire used in this study is available online at https://www.mdpi.com/article/10.3390/foods10102412/s1.
