# Supplementary material for: Food Safety Knowledge and Practices of Pregnant Women and Postpartum Mothers in Slovenia
Source: Foods. 2021 Oct 12;10(10):2412. doi: 10.3390/foods10102412 (PMC8535543; doi:10.3390/foods10102412)
Supplement: Supplementary file 1 [file foods-10-02412-s001.zip › foods-1400570-supplementary.pdf]

Questionnaire

Q1 - Gender

◯ Male
◯ Female

IF (1) Q1 ≠ [1]
Q2 - What age group do you belong to?

◯ ≤ 25 years
◯ 26-35 years
◯ 36-45 years
◯ Under 45 years

IF (2) Q1 ≠ [1]
Q3 – Marital status

◯ Married or living out of wedlock
◯ Single

IF (3) Q1 ≠ [1]
Q4 - Education

◯ Finished primary school
◯ Finished secondary school
◯ Finished higher school
◯ Finished university
◯ Finished master's/doctoral degrees

IF (4) Q1 ≠ [1]
Q5 - Place of residence

◯ Town
◯ Suburb area
◯ Village

IF (5) Q1 ≠ [1]
Q6 - Employment

◯ Student/secondary school student
◯ Part-time work
◯ Full time
◯ Unemployed/job seeker

IF (6) Q1 ≠ [1]
Q7 - You are currently:

◯ Pregnant
◯ Non-pregnant
◯ Mother after childbirth (up to 6 weeks after childbirth)

IF (7) Q1 ≠ [1]
Q8 – Number of children:

- ◯ 0
- ◯ I am currently pregnant
- ◯ 1 or 2
- ◯ 3 or more

IF (8) Q1 ≠ [1]
Q9 - In which cases do we have to wash our hands?
(Only one answer is possible.)

- ◯ After we got our hands dirty
- ◯ After using the toilet
- ◯ Before meals
- ◯ All of the above

IF (9) Q1 ≠ [1]
Q10 - How do we wash our hands properly and effectively before we start preparing food?
(Only one answer is possible.)

- ◯ Running water, soap and scrub
- ◯ Only running water
- ◯ Warm running water, soap and rubbing between fingers and around the wrists
- ◯ Warm running water and soap

IF (10) Q1 ≠ [1]
Q11 - What are the most common types of diseases that can be acquired if we do not wash our hands after using the toilet?
(Only one answer is possible.)

- ◯ Gastrointestinal diseases
- ◯ Respiratory diseases
- ◯ Psychological diseases
- ◯ Skin diseases

IF (11) Q1 ≠ [1]
Q12 - Can food and cleaning products be stored in the same place?
(Only one answer is possible.)

- ◯ Yes
- ◯ No
- ◯ I don`t know

IF (12) Q1 ≠ [1]
Q13 - Is washing fruits and vegetables important before eating?
(Only one answer is possible.)

- ◯ Yes
- ◯ No
- ◯ I don`t know

IF (13) Q1 ≠ [1]
Q14 - Where is the best place to buy safe milk?
(Only one answer is possible.)

- ◯ Milk sold in vending machines
- ◯ Sterilized and / or pasteurized milk in shops
- ◯ On farm
- ◯ All of the above

IF (14) Q1 ≠ [1]
Q15 - What is not important (from the food safety point of view) when buying canned food?
(Only one answer is possible.)

- ◯ Brand
- ◯ Vacuum packaging
- ◯ Packaging
- ◯ Durability date

IF (15) Q1 ≠ [1]
Q16 - Which of the following foods spoil most quickly at room temperature?
(Only one answer is possible.)

- ◯ Milk and milk products
- ◯ Fruit and vegetables
- ◯ Cerals
- ◯ Legumes

IF (16) Q1 ≠ [1]
Q17 - Is it safe to take during pregnancy:
(Mark only one statement per line.)

	Yes	No	Don`t know
Ready to eat meals including raw meat (e.g. tartare, carpaccio,…)	◯	◯	◯
Pastries or cakes containing raw eggs (e.g. tiramisu)	◯	◯	◯
Raw fish (sushi)	◯	◯	◯
Chateau (a dish made from raw eggs, boiled wine and sugar)	◯	◯	◯
Eggs with liquid yolk	◯	◯	◯

IF (17) Q1 ≠ [1]
Q18 – How often do you check or pay attention to… while shopping foodstuffs?
(Mark only one statement per line.)

	Never	Sometimes	Usually	Almost always	Always
… durability date.	◯	◯	◯	◯	◯
… the origin of each product.	◯	◯	◯	◯	◯
… food declaration.	◯	◯	◯	◯	◯
… content of vitamins and minerals.	◯	◯	◯	◯	◯
… hygiene of service.	◯	◯	◯	◯	◯
… food storage conditions.	◯	◯	◯	◯	◯
… refrigeration temperatures.	◯	◯	◯	◯	◯
… condition of packaging.	◯	◯	◯	◯	◯
… orderliness of the seller (e.g. clean work clothes, hair cover).	◯	◯	◯	◯	◯

IF (18) Q1 ≠ [1]
Q19 - Have you ever taken the temperature in your refrigerator at home?

◯ Yes
◯ No

IF (19) Q1 ≠ [1] and Q19 ≠ [2]
Q20 – Write down the temperature of your refrigerator at home?

[ ]

IF (20) Q1 ≠ [1] and Q19 ≠ [1]
Q21 – What do you think is the temperature of the cold air in your refrigerator?

[ ]

IF (21) Q1 ≠ [1]
Q22 - Foods brands are always of high quality.
(Only one answer is possible.)

◯ I agree
◯ I partly agree
◯ I do not agree

IF (22) Q1 ≠ [1]
Q23 - The nutritional value of a food is more important than the taste of the food.
(Only one answer is possible.)

◯ I agree
◯ I partly agree
◯ I do not agree

IF (23) Q1 ≠ [1]
Q24 - Foods sold in large stores or shopping malls are of better quality than those you buy from local producers.
(Only one answer is possible.)

◯ I agree
◯ I partly agree
◯ I do not agree

IF (24) Q1 ≠ [1]
Q25 - Free gifts offered with groceries are effective for better food sales.
(Only one answer is possible.)

◯ I agree
◯ I partly agree
◯ I do not agree

IF (25) Q1 ≠ [1]
Q26 - It is important that both, producers and traders involved in the food chain, have adequate evidence of food safety.
(Only one answer is possible.)

◯ I agree
◯ I partly agree
◯ I do not agree

Q27 - Where did you get information on food safety?
(Several answers are possible.)

- ☐ Recommendations from retailers when shopping
- ☐ Slovenian Consumer Association
- ☐ Parenting class
- ☐ Media (TV, radio, magazines, etc.)
- ☐ Doctors
- ☐ Midwives
- ☐ Other:

IF (26) Q1 ≠ [1]
Q28 - Indicate on the scale how often do you...
(Mark only one statement per line.)

	Never	Sometimes	Usually	Almost always	Always
…separate cutting boards for raw meat and raw vegetables.	◯	◯	◯	◯	◯
… wash hands after handling eggs.	◯	◯	◯	◯	◯
…wash dishes with detergent or in the dishwasher before re-use.	◯	◯	◯	◯	◯
…wash cutting board after cutting raw and before cutting heat-treated meat.	◯	◯	◯	◯	◯
… wash salad in the kitchen sink before handling raw meat.	◯	◯	◯	◯	◯
… separate cutting boards for raw and cooked food.	◯	◯	◯	◯	◯

IF (27) Q1 ≠ [1]
Q29 – Do you agree that …
(Mark only one statement per line.)

	I do not agree	I partly agree	I agree
... we are allowed to touch cooked food after contact with raw foods.	◯	◯	◯
... expired food must be discarded.	◯	◯	◯
... meat and fish are kept in the refrigerator separately.	◯	◯	◯
... ... food information and labels must be checked before consumption.	◯	◯	◯

IF (28) Q1 ≠ [1]
Q30 - Have you ever heard of the following microorganisms that are hazardous to human health?

	Yes	No
Salmonella	◯	◯
Listeria	◯	◯
Toxoplasma gondi	◯	◯
Campylobacter	◯	◯

IF (29) Q1 ≠ [1]
Q31 - Have you ever been poisoned by food?

- ◯ Yes
- ◯ No
- ◯ I do not know

IF (30) Q1 ≠ [1] and Q31 ≠ [2, 3]
Q32 - Please indicate which food was the cause of poisoning:

IF (31) Q1 ≠ [1]
Q33 – Is there an increased risk for infection during pregnancy because of…:

	Yes	No	I don't know
Listeria monocytogenes	◯	◯	◯
Toxoplasma Gondii	◯	◯	◯

IF (32) Q1 ≠ [1]
Q34 - Do you agree that …
(Mark only one statement per line.)

	I do not agree	I partly agree	I agree
… milk should be stored on the refrigerator door.	◯	◯	◯
… cooked foods should be stored at room temperature covered until they cool down.	◯	◯	◯
… hard-boiled eggs can be stored at room temperature for more than two days.	◯	◯	◯
… cooked and raw foods must be stored separately.	◯	◯	◯
... freezing food does not kill bacteria, but only stops them from multiplying.	◯	◯	◯
… different food groups need to be stored separately.	◯	◯	◯

IF (33) Q1 ≠ [1]
Q35 - How often do you prepare and eat…
(Mark only one statement per line.)

	Never	Sometimes	Usually	Almost always	Always
… pastries or cakes containing raw eggs?	◯	◯	◯	◯	◯
… ready to eat meals including raw meat (e.g. tartare, carpaccio,…)?	◯	◯	◯	◯	◯
… raw fish (e.g. sushi)?	◯	◯	◯	◯	◯
… Chateau (a dish made from raw eggs, boiled wine and sugar)?	◯	◯	◯	◯	◯

IF (34) Q1 ≠ [1]
Q36 - What is the most common way you thaw frozen foods?
(Several answers are possible.)

☐ On the kitchen counter, at room temperature
☐ In the microwave
☐ In the refrigerator
☐ In hot water
☐ Immediate heat treatment.
☐ Others:

IF (35) Q1 ≠ [1]
Q37 - Thawed meat should not be frozen agin.

◯ I agree
◯ I partly agree
◯ I do not agree

IF (36) Q1 ≠ [1] and Q7 ≠ [1, 2]
Q38 - Indicate on the scale how often ...
(Mark only one statement per line.)

	Never	Sometimes	Usually	Almost always	Always
... do you wash your hands with soap and running water after changing baby diapers.	◯	◯	◯	◯	◯
... wash your hands with soap and running water before preparing infant formula or for breastfeeding.	◯	◯	◯	◯	◯
... boil water for 2 minutes before cooling and mixing with infant formula.	◯	◯	◯	◯	◯
... do you leave the infant formula or bottled breast milk out of the refrigerator at room temperature for more than 2 hours.	◯	◯	◯	◯	◯

IF (37) Q1 ≠ [1] and Q7 ≠ [1, 2]
Q39 – What do you most often use to store pumped breast milk?
(Answer only if you are breastfeeding.)

◯ Plastic containers
◯ Glass containers
◯ Plastic bags

IF (38) Q1 ≠ [1] and Q7 ≠ [1, 2]
Q40 – Is it necessary to dispose unconsumed breast milk?

◯ Yes
◯ No
◯ I do not know
